# Psychologists’ Perspectives on the Psychological Suffering of Refugee Patients in Brazil

**DOI:** 10.1007/s11013-021-09717-6

**Published:** 2021-04-22

**Authors:** Gesa Solveig Duden, Sofie de Smet, Lucienne Martins-Borges

**Affiliations:** 1 Department of Psychology, University of Osnabrück, Neuer Graben, 49074 Osnabrück, Germany; 2 Departamento de Psicologia, Universidade Federal de Santa Catarina, Campus Universitário – Trindade, Florianópolis, SC CEP: 88040-500 Brazil; 3 Parental and Special Education Research Unit, Refugee Trauma Care-Clinical Centre PraxisP, Faculty of Psychology & Educational Sciences, University of Leuven, Leopold Vanderkelenstraat 32 bus 3764, Louvain, 3000 Belgium; 4 École de Travail Social et de Criminologie, Faculté des sciences sociales, Université Laval, Avenue des Sciences-Humaines, Quebec, G1V 0A6 Canada

**Keywords:** Refugee mental health in Brazil, Qualitative interviews, Psychiatric diagnostic manual, Depression, Anxiety

## Abstract

Worldwide there are 79.5 million displaced people, many of which face war, violence, tragic flights and struggles in host countries. Research shows augmented prevalence rates of mental disorders among refugees internationally, but little is known about refugee mental health in Latin American countries. Furthermore, only a few studies have taken into consideration the knowledge of clinical psychologists who treat refugee patients. The present study examines the experiences of 32 psychologists in Brazil regarding their refugee patients’ psychological suffering and mental disorders. Semi-structured interviews were conducted in various locations in Brazil and analysed following a consensual qualitative research approach. Four clusters of refugee patients’ suffering were synthesised: post-migration stressors, traumatic experiences, flight as life rupture, and the current situation in the country of origin. The most frequently described conditions in patients were anxiety and depression. However, the results also show that the use of manuals for the classification of mental disorders is contested among psychologists in Brazil. Most psychologists stressed patients’ socio-political suffering and saw patients’ symptoms as normal reactions to their experiences. There is a need to acknowledge the socio-political suffering of refugees in Brazil and foster their mental health by tackling current post-migration stressors such as discrimination.

## Introduction

Mental healthcare for refugee patients^1^ is an issue of international concern and is growing in importance as the number of displaced people worldwide is rising and now exceeds 79.5 million (UNHCR 2020). Refugees are very heterogenous and becoming and being a refugee is not of psychological, but of socio-political nature (Papadopoulos 2007). However, many studies find higher prevalence rates of mental disorders in refugees in comparison to the general population of host countries. For instance, rates of post-traumatic stress disorder (PTSD) have been found to be up to 10 times higher in refugees than in the general population (Fazel, Wheeler and Danesh 2005). Elevated rates of depression and anxiety^2^ have also been described in refugees (Lindert et al. 2009; Turrini et al. 2017). These findings can be explained by the psychological impacts of pre-flight exposure to war, persecution and violence, the displacement experience and post-migration struggles in host countries (Knipscheer and Kleber 2006; Miller and Rasmussen 2010).

Recently, research on refugee mental health has shifted from the concentration on pre-migration trauma towards a focus on post-migration stressors (Davidson, Murray and Schweitzer 2008; Silove, Ventevogel and Rees 2017) and on how these stressors might interplay with and exacerbate previous traumas and suffering (Cleveland, Rousseau and Guzder 2014). Post-migration stressors include experiences of culture shock and cultural bereavement (Bhugra and Becker 2005; Eisenbruch 1991; Oberg 1960), social isolation (Miller and Rasmussen 2010), discrimination (Beiser and Hou 2016), prolonged detention, tedious asylum application procedures, insecure residency status (Comtesse and Rosner 2019; Davidson, Murray and Schweitzer 2008), financial troubles (Bogic et al. 2012), limited access to services and unemployment (Silove, Ventevogel and Rees 2017).

The post-migration stressors are primarily shaped by the juristic, structural, cultural and societal conditions of the host countries which vary considerably from country to country (Davidson, Murray and Schweitzer 2008). This makes the experiences and suffering of refugees unique in each context. Furthermore, societal conditions, together with traditions in psychological sciences, influence what is considered mentally healthy or pathological (Kirmayer et al. 2015). Meanwhile, most studies investigating the mental health of refugees in host countries stem from the Global North and high-income countries. This prevalent selective geographical bias that is present in psychological research in general, leads to mental health concerns being defined by high-income countries (Kirmayer and Pedersen 2014). Some studies have looked at refugee mental health in middle- or low-income countries such as Bosnia, Uganda and Sudan (see Reed et al. 2012). In Latin American countries until 2009 few studies concerning refugees had been conducted (de Moraes Weintraub 2012; da Teixeira, Lotufo Neto and Skokauskas 2013). However, recently, with the increase in migrants and refugees, particularly from Haiti and Venezuela, more studies have focused on refugees in Latin America, such as on Colombian refugees in Ecuador (Carrasco García 2010) or in Chile (Liberona Concha and López San Francisco 2018), on Palestinian refugees in Chile (Bijit Abde 2012), on Haitian immigrants (Barros and Martins-Borges 2018) and on Syrian refugees in Brazil (Jibrin 2017; Lodetti 2018). However, there remains a need to study concepts of pathology, in particular related to the mental health of refugees, in a greater variety of cultures and contexts (Duden & Martins-Borges 2021) and to insert such studies and their findings into an (Anglophone-dominated) global research world, in order to call international attention to these studies and findings from diverse contexts. Following the suggestion of an inside-out model that prioritises the perspectives of those underrepresented in research (Gergen, Josselson and Freeman 2015; Hall, Yip and Zárate 2016; Syed et al. 2018), the present study aimed to look at the perspectives of psychologists who treat refugees in Brazil.

In Brazil, by June 2020, the total number of recognised refugees exceeded 43,000 of which about 38,000 are Venezuelans (Agência Brasil 2020; CONARE 2020). About 80,000 new applications were submitted in 2018 alone (CONARE 2020). This represents a considerable increase in asylum applications in Brazil, inter alia related to the earthquake in Haiti in 2010 and the humanitarian crisis in Venezuela from 2016 onwards. Brazilian jurisdiction relies on a comparably broad definition of “refugee” and also allows the possibility of humanitarian visa (Bógus and Rodrigues 2011; Patarra and Fernandes 2011). In November 2017, the Brazilian state approved the “nova lei de migração”—a new law for migrants, that aims to simplify administrative procedures for immigrants in Brazil. It shifts from a perspective of migrants as risks for national security to a human rights perspective, an emphasis on the guarantee of rights and non-discrimination of migrants (Assis 2018). Among other things, the new law facilitates the passage of by-laws for the concession of humanitarian visa (de Oliveira 2017). Meanwhile, the new law did not pass without opposition. For instance, according to Assis (2018:619), the veto against the use of the term “migrant” in the law (the new law includes only the definitions of “immigrant”, “emigrant”, “border resident”, “visitor” and “stateless person”) expresses the persistence of aiming to place migrants closer to the idea of strangers and foreigners, instead of fostering a perception of migrants as mobile subjects in the contemporary world, which was what the commission originally set out to establish. Still, there are various notable aspects in the Brazilian jurisdiction for migrants, such as that asylum seekers are granted a work permit directly after applying for asylum, have the same rights to access education, training and healthcare as Brazilians (Assis 2018) and that there is no deportation in Brazil in general (Jubilut 2006; Leão 2011).

The present study aimed to investigate the experiences of psychologists regarding their refugee patients’ psychological suffering. We decided to interview professionals as experts due to our assumption that psychologists who encounter refugees in their daily practise might have special insight into the mental health and suffering of their patients (Meuser and Nagel 2009). Investigating their experiences and knowledge might be a valuable first step for understanding refugee patients’ situation in Brazil and develop appropriate mental healthcare services. We focused on professionals in Brazil for two main reasons: Firstly, even though there has been a recent increase in studies investigating various aspects of refuge in Brazil (Duden & Martins-Borges 2021; Barros and Martins-Borges 2018), there is still little information concerning their mental health across several Brazilian states (Braga Bezerra, Martins-Borges and Cunha Pereira 2019; de Moraes Weintraub 2012; da Teixeira, Lotufo Neto and Skokauskas 2013), and even more so, a lack of international attention to this specific field of research. Secondly, gaining the perspectives of experts from a country from the Global South might provide new insights for the discussion on the categorisation of mental disorders (Drodžek 2007; Kirmayer 2001; Summerfield 1999, 2008). Our research design addressed participants’ perspectives as culturally mediated phenomena located in the Brazilian context, with the aim to understand “explanatory models” of practitioners (Kleinman 1980). This concept from medical anthropology stresses the importance of causal attributions and ethnophysiological theories on illness experience, but also on treatment response (Kirmayer and Sartorius 2007; Watters 2001). In other words, how psychologists in Brazil think about the suffering of their refugee patients will influence how they attend to the needs of those patients within the Brazilian context.

Specifically, we formulated the following research questions:

How do psychologists who work with refugees in Brazil experience the psychological suffering of their patients?

- How do they describe the suffering?
- Which mental health disorders and symptoms do they perceive to be most common among their refugee patients?

## Method

### Study Design

This article forms part of a larger study investigating Brazilian approaches to the mental healthcare of refugees. The focus of the present investigation was how psychologists experience the psychological suffering of their refugee patients. In order to allow for an in-depth analysis of psychologists’ perspectives located in the Brazilian socio-political and cultural context, the research project used qualitative procedures of inquiry resting on a constructivist ontology and a subjectivist epistemology. The study consisted of semi-structured interviews which were analysed adopting a consensual qualitative research (CQR; Hill et al. 2005) approach.

### Participants and Procedures

Gatekeepers and contacts to local NGOs were used for recruitment, as well as subsequent snowball sampling. The Ethics Committee of the University of Osnabrück gave ethical approval for the study and informed written consent was obtained from all participants. A semi-structured interview guideline was developed with the objective of encouraging participants to speak about their perspectives on their refugee patients’ suffering. It included questions on common characteristics of participants’ patients, such as patients’ country of origin (CoO), questions on patients’ psychological suffering, symptoms and mental health disorders as well as on psychologists’ use of diagnostic manuals and categorical classification of mental disorders (MDC). The latter was incorporated to allow for a contextualisation of the findings on frequent disorders, as we assumed that usage of MDC, such as for instance the DSM-5, would influence the way psychologists spoke about patients’ suffering. The guideline was pilot tested with Brazilian psychologists to ensure the unambiguity of questions. Between November 2018 and May 2019, semi-structured interviews were conducted with participants (*N* = 32) who held a university degree in psychology and had been working with refugee patients for at least 6 months (Table 1). 44% of the psychologists were of psychoanalytic orientation, reflecting the often-reported predominance of this approach among general clinical psychologists in Brazil (González 2018; Yamamoto 2012). Eight participants worked with Ethnopsychiatry which is an encounter and complementarism of psychoanalysis and anthropology (Martins-Borges et al. 2019; Nathan 2001). These participants had also taken part in courses of “cultural psychology” during their academic studies and trained in university clinics to attend refugee patients psychologically. None of the other participants had received any specific training to work with survivors of trauma, refugees or to work transculturally, but six of the participants had a migrational background. Most of the participants did not exclusively attend refugee patients, but also worked with other immigrants and Brazilians.

**Table 1:** Participants’ characteristics

Characteristic (*N* = 32)	*N* (%) or *M* (SD)	Range
Age (years)	34.76 (10.73)	23–61
Experience working with refugees (years)	3.88 (3.9)	1–20
Region of work		
North	6 (19)	
Southeast	7 (22)	
South	19 (59)	
Gender		
Women	25 (78)	
Men	7 (22)	
Professional approach		
Cognitive–behavioural	2 (6)	
Community and social	4 (13)	
Ethnopsychiatry	8 (25)	
Existential and humanistic	3 (9)	
Psychoanalytic-dynamic	14 (44)	
Systemic	1 (3)	
Origin		
Argentina	1 (3)	
Brazil	26 (81)	
Columbia	1 (3)	
Lebanon	1 (3)	
Peru	1 (3)	
Syria	1 (3)	
Uruguay	1 (3)	
Working languages^a^		
Portuguese	32 (100)	
Spanish	22(68.8)	
French	6 (18.8)	
English	11 (34.4)	
Arabic	2 (6.3)	
Attending refugees in^a^		
Refugee housing	5 (15.6)	
University clinic	13 (40.6)	
Private practice	3 (9.3)	
NGO setting	12 (38)	
CRAI^b^	3 (9)	

Data are total number (percentage) or mean (standard deviation) and range

^a^Multiple responses possible

^b^Psycho-social Centre for Refugees

Interviews lasted between 40 min and 2 h. They were conducted in Portuguese, either face-to-face at participants’ workplaces (*n* = 19) or via skype (*n* = 13), audio recorded and fully transcribed. To secure the anonymity of participants, all identifying information was removed, the names of participants were replaced for codes and locations of practice were collated into regions.

### Data Analysis

Interview transcripts were analysed focusing on the overt meaning in the data, i.e. what is said and by whom. CQR (Hill et al. 2005) was adopted as this approach represents a systematic method to evaluate the representativeness of issues across cases. First, every interview was read thoroughly and sections related to psychological suffering of refugees were highlighted. The first author coded highlighted sections line-by-line and developed a hierarchical coding tree by collating the codes into subthemes, themes and categories. In order to assess the fit of the coding tree, the first author and two further researchers re-applied it to the interview transcripts independently and subsequentially discussed until a consensus about the codes, subthemes, themes, and their hierarchical structure was found. These steps were carried out in Portuguese (González and Lincoln 2006). The coding tree was then translated into English, using a bilingual committee (Brislin 1970). Next, the whole data set was organised into the English coding tree using the qualitative analysis software MAXQDA (VERBI-Software (2020). MAXQDA 2020 [computer software]. Berlin, Germany.: VERBI Software. Retrieved from https://www.maxqda.com^2020^) in order to systematically represent the frequencies of participants endorsing each theme and subtheme (Tables 2, 3). The frequency counts enable an overview of the presence of each theme in the data set (Malterud 2001). Themes were labelled “general” if they were endorsed by 31–32 participants, “typical” if they applied to 16–30 cases, “variant” for less than 16 but at least 5 cases, and “rare” if endorsed by only 1 to 4 participants (Hill et al. 2005).

### Researchers’ Positionality

The study’s principal investigator who conducted the interviews and served as the primary coder is a PhD candidate in cultural psychology. Being of German origin, she gained awareness for challenges in post-migration settings through her own experiences of migration and academic interests in refugee studies. The fact that she is of German nationality might have, in regard to the history of colonisation (González and Lincoln 2006), impacted the extent to which participants felt at ease to articulate their perspectives. Yet, the principal investigator had worked for an extended period of time among psychologists in Brazil and was, thus, familiar with styles of communication and interaction in this context. Moreover, the other two coders of the study were Brazilian clinical psychologists which improves the likelihood that the manner in which psychologists in Brazil speak about psychological suffering was captured accurately. All three researchers have extensive experience using qualitative methods of inquiry. Informed by the literature on refugee mental health, the researchers expected participants to highlight PTSD, depression and anxiety as common mental health problems in their patients.

## Results and Discussion

**Table 2:** Categories of suffering

Category	Theme	Subtheme	Frequency (P/C)	Region
Post-migration situation	Intolerance, hostile attitudes and violations	Xenophobia	Variant (14/23)	All
		Discrimination	Variant (15/18)	All
		Racism	Variant (12/15)	S, SE
		Not being recognised by society	Variant (10/14)	S, SE
		Rights violations	Variant (10/14)	N, S
		Being objectified as refugee	Variant (6/10)	S, SE
		LGBTI-phobia	Rare (4/6)	N, S
	Isolation	Loneliness	Variant (12/26)	All
		Family separation	Variant (14/24)	All
		Feeling of not belonging	Variant (9/17)	All
		Fragile networks	Rare (2/2)	S
	Cultural adaptation	Cultural shock	Variant (15/23)	All
		Conflicts with own culture	Variant (10/21)	S, SE
		Difficulties of integration	Variant (13/25)	All
		Homesickness	Variant (6/9)	N, S
	Precarious situation	Precarious economic situation	Typical (22/44)	All
		Insecurity of future	Variant (7/11)	All
		Dependency and helplessness	Variant (13/22)	S, SE
		Hunger and thirst	Variant (12/18)	All
		Living on street	Variant (11/15)	N, S
		Other housing problems	Variant (9/13)	All
Traumatic experiences	Violence	Sexual violence	Variant (11/18)	All
		Domestic violence	Variant (10/15)	All
		Other violence	Variant (15/20)	All
		Torture	Rare (1/1)	S
	War experiences		Variant (6/8)	S
	See people dying		Variant (7/7)	S, SE
	Being persecuted		Variant (5/7)	S, SE
	Robbery		Variant (6/10)	All
	Surviving an earthquake		Variant (6/6)	S, SE
	Victim of human trafficking		Rare (4/5)	N, S
	Crossing the Amazon forest		Rare (4/5)	S
Flight as life rupture	Flight as involuntary		Typical (17/31)	All
	Flight as existential crisis		Rare (4/5)	S
	Experiences of loss	Generically mentioned^a^	Typical (23/73)	S, SE
		Loss of social function	Variant (13/20)	All
		Loss of culture and language	Variant (11/23)	All
		Loss of relations	Variant (10/11)	All
		Loss of identity	Variant (5/9)	All
		Loss of place	Variant (5/5)	All
		Material loss	Rare (2/2)	S
Current Situation in CoO	Expectations from family		Variant (8/14)	All
	Unstable CoO		Variant (5/15)	All
	Guilt of surviving		Variant (6/8)	All
	No reparation for violations		Rare (2/2)	S

*CoO* country of origin, *N* North, *S* South, *SE* Southeast

^a^“Generically mentioned” refers to the cases where participants mentioned the theme without specifying it in a subtheme

**Table 3:** Disorders and symptoms mentioned by psychologists organised according to the DSM-5 (American Psychiatric Association 2013)

Disorders	Section	Symptoms	Frequency (#P)	Region	Total (#P)^e^
Depressive disorders	Diagnosis^a^: Major Depressive Disorder	–	Variant (11)	All	Typical (22)
	Symptoms, generic^b^	–	Variant (8)	N, S	
	Symptoms, specific^c^	Sadness	Variant (8)	All	
		Suicidal ideation	Variant (7)	All	
		Tearfulness/crying	Variant (6)	All	
		Diminished interest/apathy	Rare (3)	N, S	
		Social withdrawal	Rare (1)	S	
		Poor concentration	Rare (1)	S	
		Low self-esteem	Rare (1)	S	
		Fatigue	Rare (1)	S	
		Hopelessness	Rare (3)	N, S	
Anxiety disorders	Diagnosis: Generalized Anxiety Disorder	–	Variant (11)	N, S	Typical (17)
	Symptoms, generic	–	Variant (6)	S	
	Symptoms, specific	Excessive fear	Rare (3)	N, S	
		Panic attacks	Variant (6)	All	
Posttraumatic stress disorder (PTSD)	Diagnosis: PTSD	–	Variant (5)	All	Variant (10)
	Symptoms, generic	–	Rare (3)	All	
	Symptoms, specific	Dissociative reactions (flashbacks)	Rare (3)	S	
Somatic symptom and related disorders	Diagnosis	–	–	–	Variant (7)
	Symptoms, generic	–	Variant (5)	N, S	
	Symptoms, specific	Abdominal pain	Rare (3)	S	
		Headache	Rare (3)	S	
		Backache	Rare (1)	S	
		Chest pressure	Rare (1)	S	
Schizophrenia spectrum and other psychotic disorders	Diagnosis: Psychotic Disorders	–	Rare (4)	S, SE	Variant (6)
	Symptoms, specific	Delusion of persecution	Rare (1)	S	
		Hearing voices	Rare (1)	N	
Substance-related and addictive disorders	Diagnosis: Generic	–	Rare (3)	All	
Autism spectrum disorder	Diagnosis	–	Rare (1)	N	
Adjustment disorder	Diagnosis: Adjustment Disorder	–	Rare (1)	N	
Other categories and symptoms^d^		Sleep disturbances	Variant (5)	N, S	
		Aggressivity	Rare (1)	S	
		Impulsivity	Rare (1)	S	
	Grief		Variant (12)	All	(12)
	Severe Stress		Variant (5)	N, S	(5)

^a^The term “diagnosis” is indicated when psychologists explicitly mentioned the respective diagnostic category

^b^The term “generic” is indicated to show the number of psychologists mentioning general symptoms of a disorder (e.g. “depressive symptoms”) rather than specifying them further

^c^“Specific” refers to the specific symptoms of a disorder described by participants

^d^Categories and Symptoms which are either not listed in the DSM-5, but were highlighted by participants, or which were not mentioned uniquely in relation to one specific disorder

^e^The total refers to the sum of participants who referred to specific symptoms and/or the diagnostic category itself

### Refugee Patients’ Backgrounds

**Fig. 1:**
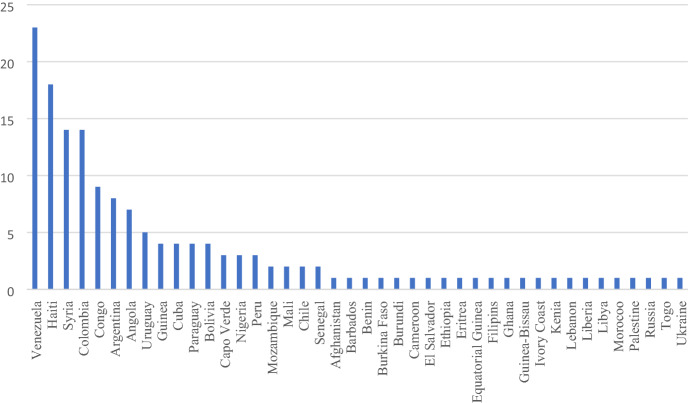
Number of psychologists mentioning the respective country of origin of their (migrant and/or refugee) patients

In order to contextualise the findings on patients’ suffering, psychologists were asked to name the country of origin (CoO) of their patients as well as additional characteristics they deemed important. In doing so, participants did not distinguish between patients’ official immigration or asylum status. Thus, the CoOs reported here are of diverse immigrants, including refugees and asylum seekers. In sum, participants named 42 different CoOs (see Figs. 1). Most frequently mentioned CoOs were Venezuela, Haiti, Syria, and Colombia. Other frequently mentioned characteristics of patients were pregnant women or single mothers, lesbian, gay, bisexual, transgender, and queer people.

**Fig. 2:**
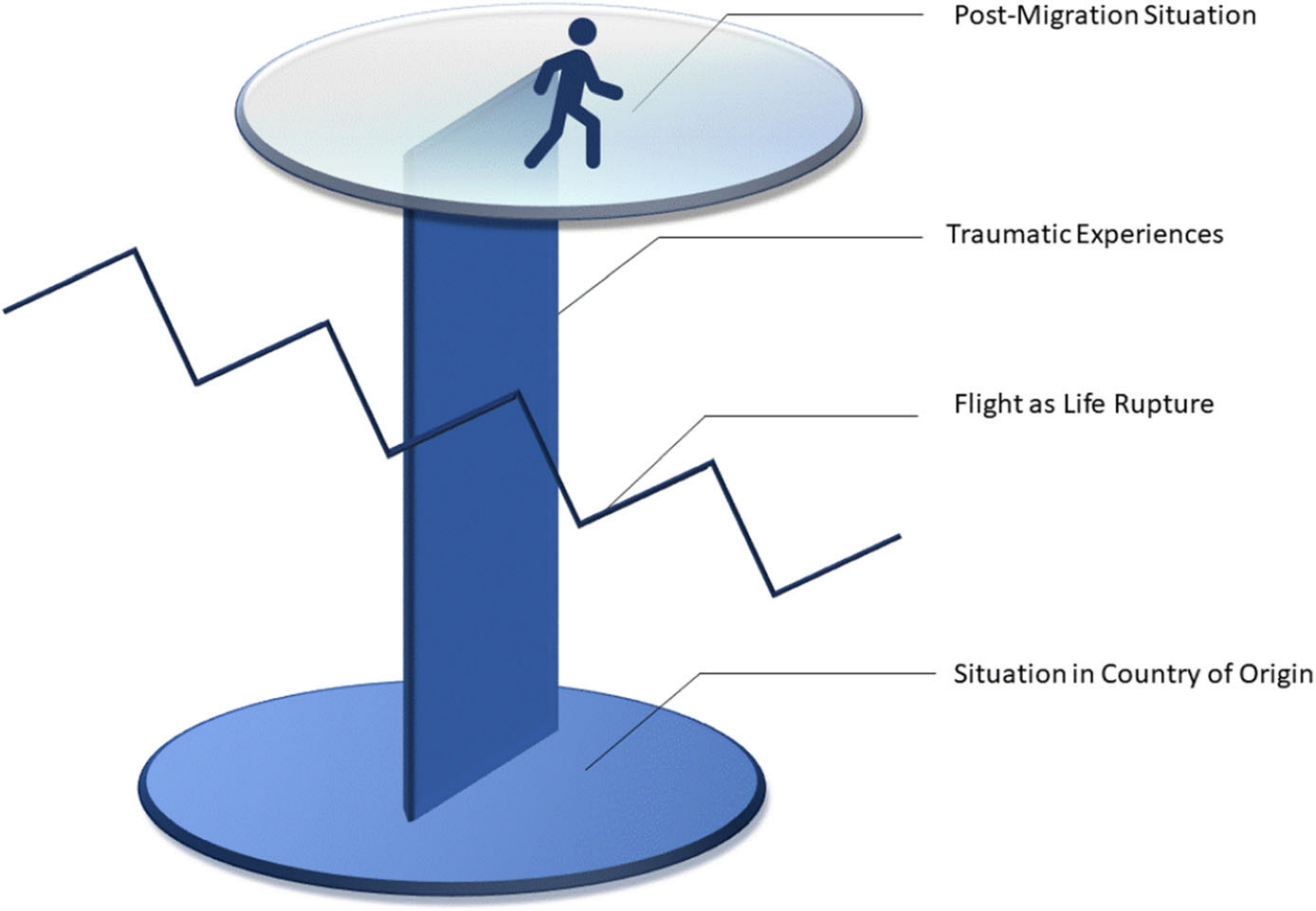
Categories of Suffering. Traumatic experiences are represented as transcending the CoO, the flight as well as the post-migration situation

### How Do Psychologists Describe the Suffering of Their Refugee Patients?

The following section synthesises in four categories what psychologists perceived to constitute the psychological suffering in their refugee patients (Table 2). Figure 2 displays these categories as interlinked: Currently, the refugee finds herself in a context of **post-migration difficulties** (upper part of the figure), her life was **ruptured by the flight** (zig-zag-line), the **situation in the CoO** is still present for her (forming the base of the figure) and **traumatic experiences** transcend the CoO, the flight as well as the post-migration situation.

#### Post Migration Situation

All participants stressed how the post-migration context causes substantial suffering in refugee patients. This category took up the most considerable portion of all interview transcripts. The following factors were perceived as particularly impactful for patients:

### Hostile Attitudes, Intolerance and Rights Violations

There is suffering for not being recognized by the other. We have many refugees here in Brazil who are black, who come from African countries or Haiti. Blacks suffer a lot from racism. Racism is very strong here. They tell me: “What do I do if I walk on the street, people start walking very fast, they don't stay on my side.” That creates suffering. (P15, psychoanalytic, Age: 41, South)

All participants mentioned that their patients were suffering from experiencing hostile attitudes and intolerance, in particular by being subject to xenophobia, discrimination, racism, LGBTQ-phobia, as well as not feeling recognised by society or objectified as a refugee. Variant participants also described the human rights violations their patients were experiencing in Brazil, including physical mistreatment, (sexual) abuse and slavery work.

Discrimination and xenophobia have been described as major obstacles for the integration of refugees in Brazil and as strongly related to the struggle of finding employment (Moreira and Baeninger 2010). Internationally, experiencing discrimination in host countries has been found to greatly impact the mental health of refugees (Bogic et al. 2012), sometimes even more than pre-migratory trauma (Beiser and Hou 2016).

### Isolation

Isolation was another factor participants typically saw as part of the suffering of patients: “[…] a state of complete loneliness. She feels disconnected from people. She feels that no one will understand her. And she feels that she won't understand anyone” (P9, ethnopsychiatric, Age: 28, South). Psychologists described patients’ feelings of loneliness and not-belonging and highlighted the fact that patients often suffered due to being separated from their families. Two participants mentioned that refugee patients had very fragile networks in their new context.

Social isolation has been described as a major stressor in post-migration settings (Miller and Rasmussen 2011). Eisenbruch (1991) integrated the loss of social networks and of a sense of belonging in the concept of “cultural bereavement” which he considered characteristic of forced displacement (e.g. Bhugra and Becker 2005). Reinforcing this, others have found associations between the wellbeing of displaced people and the reconstruction of social networks and a feeling of community (Summerfield 1999).

### Cultural Adaptation

As a further variant factor of suffering in the post-migration situation, processes of cultural adaptation were described. Participants mentioned the patients’ struggles with integrating into Brazilian society, experiencing cultural shocks and finding it hard to build relationships with Brazilians: “They report a lot of difficulty in establishing ties with people here” (P23, psychoanalytic, Age: 60, Southeast). Together with feelings of homesickness these struggles contributed to the sense of isolation in patients. Furthermore, psychologists saw some of their patients as coming into conflict with their culture of origin.

The latter may, as research suggests, be particularly impactful in individuals with families and cause intergenerational conflicts since family members can differ in their speed and way of acculturating (Droždek 2007; Leyendecker et al. 2018). There have been inconsistent results in the literature regarding the relationship of acculturation and mental health (Kartal and Kiropoulos 2016), but often cultural adaption is viewed as a cause of substantial distress in immigrants (Knipscheer and Kleber 2006; Martins-Borges 2013, 2017).

### Precarious Situation

The precarious nature of the patients’ context was typically perceived as a further aspect of refugees’ suffering: “the precariousness of his life made him feel paralyzed and unable to act” (P10, community/social, Age: 27, South). Variant participants described patients fearing for basic needs, experiencing hunger or living on the street. Furthermore, participants typically highlighted the economic instability and financial troubles of patients due to a lack of stable employment and financial support structures. Variant psychologists mentioned that the precarious situation evoked feelings of helplessness and humiliation in many refugees. Participants also stressed the impact of future insecurity on patients:

The greatest suffering is the uncertainty. They are not “refugees” when they arrive. They are ‘asylum seekers’, a totally preliminary and provisional situation that can last three to five years. This reinforces the liminal character of migration... being in and out... which makes it impossible to build long-term plans. (P3, community/social, Age: 61, Southeast)

Even though there is no detention and deportation in Brazil (Jubilut 2006; Leão 2011) and these two aspects have been most strongly associated with future insecurities (Davidson, Murray and Schweitzer 2008; Momartin et al. 2006), participants described strong feeling of insecurity in their refugee patients. These feelings can be understood in light of a context in which refugee applications are only processed slowly, in which high rates of extreme poverty and social inequality are common (de Barros, Henriques and Mendonça 2001) and in which refugees struggle with unmet basic needs and a lack of integration structures (Knobloch 2015; Moreira and Baeninger 2010). For instance, Aydos, Baeninger and Dominguez (2008) reported that about 37% of displaced people lived on the street after arrival in Brazil. A strong association between social inequality, poverty and mental health disorders has been stressed for the general population in Brazil (Silva and Santana Santana 2012). Concerning refugees, employment and economic stability have been found highly important for good health outcomes (Bogic et al. 2012; Lindert et al. 2009; Knipscheer and Kleber 2006). The effect of future instability due to tedious asylum procedures and temporary visa on the mental health of asylum seekers is an issue of international concern (Comtesse and Rosner 2019; Davidson, Murray and Schweitzer 2008).

#### Traumatic Experiences

Most participants talked in some form about traumatic experiences in relation to the suffering of their patients. Patients’ traumas included experiences of war, surviving an earthquake, violence and human trafficking, seeing people dying, being persecuted, robbed, and crossing the Amazon forest:

She entered the Amazon forest and spent days on a boat in a river with crocodiles and with others who assaulted her. In this country, on her first stop after Cuba, she worked in slavery-like conditions, she was even locked-in at her work, and she had to escape. (P12, ethnopsychiatric, Age: 27, South)

The types of trauma experienced depended on patients’ trajectories and their CoO. For instance, psychologists who attended mainly Haitians often described the impacts of surviving an earthquake.

#### Flight as Life Rupture

A common issue is grief. Grief in relation to the country, to the culture, to the recognition of identity. To their life project, that was abruptly interrupted. (P24, ethnopsychiatric, Age: 33, South).

Most psychologists perceived a life rupture due to the involuntary flight as part of the suffering of their patients. The flight came with many losses that sometimes lead to the experience of existential crisis.

Leaving one’s home involuntarily without preparation for the ruptures that this flight will cause has been described as a risk factor for mental health (Martins-Borges 2013). Research has shown that experiencing loss, such as of one’s home and the sense of social belonging and connection to a land and its symbols is associated with refugees’ mental health outcomes (Davidson, Murray and Schweitzer 2008). The loss of social belonging also forms an essential element of the concept of cultural bereavement (Droždek 2007; Eisenbruch 1991).

#### Current Situation in Country of Origin (CoO)

Finally, psychologists suggested patients’ suffering was dependent on what was happening in their CoO. First, they linked patients’ distress to the expectations from relatives that remained in the CoO. Second, patients suffered when the situation in their CoO was still unstable, dangerous and difficult for those who had stayed: “First of all there is the family left behind in Venezuela. I hear a lot that it’s like a type of guilt: ‘I am here eating, I am here sleeping, and my family is starving on the street’” (P22, psychoanalytic, Age: 43, Southeast). Some patients were described to be constantly worrying about the well-being of their relatives in the CoO and to be feeling guilty for surviving and leaving people behind. Furthermore, two psychologists saw the fact that patients did not receive reparations from their CoO for violations they had experienced as impeding the healing process.

Many of the CoOs of participants’ patients, such as Venezuela and Haiti, continue in precarious situation of violence, famine and misery (de Oliveira, Yazdani and Gomes 2019). The literature shows that refugees from countries whose conflicts are still unresolved have worse mental health outcomes (Porter and Haslam 2005) and a sort of “survivor guilt” has been reported as a common phenomenon among refugees (Eisenbruch 1991; Martins-Borges 2013).

#### Which Mental Health Disorders and Symptoms Do Psychologists Perceive to Be Most Common Among Their Refugee Patients?

**Fig. 3:**
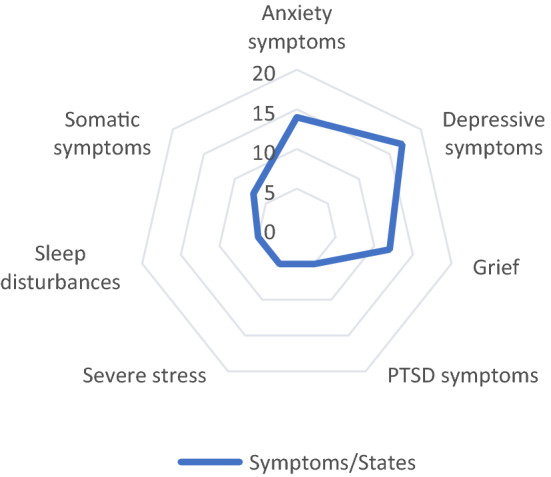
Most frequently mentioned symptoms or states. The number indicates the total of psychologists who referred to the respective symptom or state

The following section focuses on the mental disorders and symptoms participants most commonly perceived in their patients (see Table 3; Fig. 3). However, and in order to contextualise these results, we first report on whether and in which way participants made use of mental disorder classifications (MDC).

### Use of Mental Disorder Classifications

Prevailing among participants were critiques of MDCs with 18 stating they did not use psychiatric diagnostic manuals such as the DSM-5 at all.

I don't think diagnostics have any function. Because they're Western diagnostics. If I am talking about understanding the constitution of a subject in a cultural dimension, the way he suffers, and what he presents as suffering, is also a narrative of his culture. And who made the diagnoses were Westerners. From a biomedical perspective. (P6, ethnopsychiatric, Age: 28, South)

I don't work with diagnostics at all. I think they suit the pharmaceutical industry a lot because they frame the subject by his symptom […] that human part of medicine, in the case of these diagnostic manuals, has been lost. Psychiatrists, for example, have become prescribers of pain medication. There is no ‘listening’ anymore. (P17, psychoanalytic, Age: 47 years, South)

Certainly, these opinions might also reflect the fact that of the participants 44% followed psychoanalytic/-dynamic, 25% ethnopsychiatry and 16% community, social or systemic approaches (Table 1)—therapeutic orientations which do not place their emphasis on diagnostic categories (e.g. Guzder 2014; Wolitzky 2020). Some psychologists indicated the use of diagnostic strategies, but rather than applying categorical manuals such as the DSM-5, they were oriented towards psychoanalytic structural diagnostic or the axis of DSM-IV. These approaches seemed more flexible, continuous and more appropriate to them. The participants who made use of manuals such as the DSM-5 (*n* = 14), indicated employing them carefully and mostly in order to communicate with other professionals. Participants showed themselves aware of cultural and languages differences that might cause wrongful diagnosis, but they did not see this as a difficulty. Rather participants stressed that these differences made the careful and flexible use of diagnostic manuals even more important. This finding was especially pronounced in the participants who had studied cultural psychology and worked with ethnopsychiatry; meanwhile it was also represented among other participants.

A critical use of Western psychiatric diagnostic manuals is not uncommon in Latin American countries (Parra 2013). Internationally, critiques of these manuals have been made, especially concerning their universal applicability (Droždek 2007; Summerfield 1999), since the manuals themselves represent cultural products (Gone and Kirmayer 2010) and their use can lead to miss-interpretation of suffering (Kizilhan 2006; Wohlfahrt and Zaumseil 2006). Some have also posed the more general question if MDC necessarily leads to an improvement in our understanding of effective interventions (Kirmayer and Sartorius 2007). In line with many of the participants of the present study, Aveline (2005:158) argues, that the categorical perspective of psychological suffering often does not fit the “problems in living” of patients. To a large extent the interviewed psychologists embraced the idea that the focus on classified disorders was insufficient to guide their practice, as patients struggled with the “social suffering” discussed in the previous section, i.e. contextual predicaments stemming e.g. from experiencing racism, not necessarily from psychiatric disorders (Guzder 2014; Kleinman, Das and Lock 1997).

I haven’t identified depressive cases. The point is, there are cases of sadness, but if you do a little more analysis, you realize that it’s a sadness of the whole process they are experiencing in their lives at the moment. (P16, community/social, Age: 29, South).

Alternative strategies for making sense of the suffering of patients in transcultural encounters have been proposed in the literature, such as the DSM-5 Cultural Formulation Interview (APA 2013) or the McGill Illness Narrative Interview (Groleau, Young and Kirmayer 2006). This latter interview aims to elicit patients’ own narratives of their suffering in order to find pathways to care that are in line with their experience (Groleau, Young and Kirmayer 2006).

### Mental Health Disorders and Symptoms

Despite critiques of MDCs, all psychologists participated in a description of the most common symptoms and disorders of their refugee patients. The descriptions are elaborated here and represented in Fig. 3 and Table 3.

#### Depressive Disorders and Symptoms

Typically, psychologists mentioned depressive disorders and symptoms as common among their patients. Described depressive symptoms included constant crying, sadness and apathy. Three participants also referred to a deep despair in patients which they perceived as an existential hopelessness. Furthermore, suicidal ideation was often mentioned in combination with depression.

Internationally, depression has been reported with high prevalence rates among refugees (Lindert et al. 2009). In Brazil, a study of Haitian immigrants yielded that depressive symptoms were in the clinical range in 10.6% of participants (Brunnet et al. 2018). A study of Bolivian immigrants in São Paulo indicated the high probability of a mental disorder in more than half of the participants, with depressive and anxiety symptoms having the highest prevalence (Bustamante Ugarte et al. 2019).

However, many participants of the present study questioned the distinction between depressive disorder and grief. 12 of the interviewed psychologists explicitly stated they preferred to talk in terms of grief as opposed to depressive disorder and to consider their patients’ mental states, which resembled depressive phases, to be normal reactions to the many losses the refugees had faced.

The refugee suffers from grief. From a succession of bereavements. The refugee’s suffering revolves around loss. The loss of a known place, the loss of a group of people, of relationships, the loss of a social function. So, I think in terms of psychic suffering the most striking one is loss and grief. (P9, ethnopsychiatric, 28 years, South)

In the literature there has been a resurgence of interest in the construct of grief, especially concerning refugee patients (Comtesse and Rosner 2019; Momartin et al. 2004; Silove, Ventevogel and Rees 2017), since loss is such a common experience among refugees and linked to their mental health outcomes (Davidson, Murray and Schweitzer 2008).

#### Anxiety Disorders and Symptoms

Typically, psychologists also described anxiety symptoms and disorders as common in their refugee patients:

The most common psychological suffering.... Very high anxiety. Depression. Absurd. Panic attacks. […] Anxiety and panic because they find themselves in a dangerous situation and are always alert. And that means stress as well. They are always afraid that somebody could rob them, the family is starving and in need of them in Venezuela. (P1, existential/humanistic, Age: 31, North)

Research has found heightened levels of anxiety in asylum seekers (Silove et al. 1997). Lindert, et al.’s (2009) meta-analysis showed a combined anxiety prevalence rate of 40% in refugees and more recently, Turrini et al. (2017) reported that anxiety and depression in refugees were at least as frequent as PTSD and affected on average one out of three refugees. Presumably, anxiety states result from refugees’ past experience, but also from the insecurity and instability in their postmigration situation: “Anxiety disorders are very present. But for complex reasons, not for certain permanent core beliefs, but due to the external stress that is permanent” (P20, cognitive–behavioural, 43 years, South). In the case of Brazil, one might question if heightened anxiety is specific to refugees or also common in the general population and a reflection of a widespread sense of political, economic and personal insecurity. For instance, in São Paulo anxiety disorders were found to affect almost 20% of the general population (Andrade et al. 2012). Meanwhile, the study by Andrade et al. used criteria of the DSM-IV which were criticized by many participants in the present study. Furthermore, it is likely that there are enormous differences in the anxiety rates of the general Brazilian population depending of the location of research—São Paulo, the most populous city of Brazil and South America might differ considerably in this aspect compared to a rural region in Minas Gerais, for instance.

#### Posttraumatic Stress Disorder (PTSD)

Ten participants spoke of PTSD or related symptoms among their patients: “There's posttraumatic stress which we end up working with a lot. You see a lot of it. Posttraumatic stress due to displacement, due to violence” (P1, existential/humanistic, Age: 31, North). At the same time, 29 psychologists used the word “traumatic” when describing their patients’ experiences: “Many were raped and robbed on their way. They got here only with their cloths on their body - the experience from there to here is traumatic for almost everybody” (P29, psychoanalytic, Age: 23, North).

The finding that most participants described refugees’ traumas but relatively few mentioned PTSD might, firstly, be due to an epistemological difference in participants’ concepts of trauma and the concept underlying the PTSD diagnosis. When participants were invited to explain their understanding of trauma, most psychologists defined it as an event that did not have a psychic representation and was “unbearable to tell”. This demonstrates a psychoanalytic understanding of trauma (Levine 2014). A second explanation might be that, even though, robust associations have been made between pre-flight trauma and the mental health of refugees (Davidson, Murray and Schweitzer 2008; Silove et al. 1997) and refugees show higher rates of PTSD than the general population (Fazel, Wheeler and Danesh 2005), most people who have survived traumas do not develop PTSD (Bisson et al. 2015). Furthermore, traumas linked to profound losses might, in the long term, rather evoke depressive than PTSD symptoms (Momartin et al. 2004). Finally, a closer analysis of the transcripts showed that, in relation to the aforementioned critical attitude towards MDC, many participants were reluctant to use the PTSD category as they worried that this would reduce patients’ suffering to past events and turn it into an individualised and “medical” problem, thus neglecting its socio-political dimension: “Of course, trauma means that the subject has experienced a terrible traumatic scene. I'm not saying otherwise. I am saying that the socio-political situation promotes trauma and is something that stifles the subject in various ways” (P27, psychoanalytic, Age: 33, Southeast).

As with most diagnostic categories, the concept of PTSD is a product of Euro-North-American culture, context and history (Kirmayer et al. 2015), but the discourse around it has reached diverse cultural realties globally (Argenti-Pillen 2000). One might wonder if participants’ critique of the concept of PTSD, i.e. implying an individualisation of trauma, could be related to the recent history of dictatorship in Brazil which involved experiences of collective traumas (Kevers et al. 2016). However, the argument, that PTSD is too much focused on past traumas and disregards the socio-political context in which traumas occur, has been used internationally, too, as one aspect of the considerable critique of PTSD (Droždek 2007; Kevers et al. 2016; Summerfield 1999, 2008). Some authors state this focus implies neglecting the complexity of the refugee situation and missing out on the opportunity to better mental health outcomes in refugees by changing present conditions in host countries (Watters 2001). This was also stressed by the participants in the current study who, in line with Cleveland, Rousseau and Guzder (2014), consistently brought up the present post-migration situation as interplaying and exacerbating past traumas:

In the case of the Syrians, we think that we will work with war traumas. But none of them ask for trauma care in the sense we imagine. All of them have other challenges, especially the daily challenge of living as a refugee here in Brazil. (P19, systemic, Age: 30, South)

#### Other Mental Disorders and Symptoms

Other mental health conditions described by more than one psychologist included somatic symptoms, psychotic disorders and symptoms, severe stress, sleep disturbances and substance abuse: “There are a lot of cases of alcoholism, in women too” (P30*,* existential/humanistic, Age: 40, North).

Most of these disorders and symptoms have been described in previous studies concerning the mental health of refugee patients (Silove, Ventevogel and Rees 2017). For instance, a number of studies has linked PTSD symptoms to psychotic symptoms in refugees (Nygaard, Sonne and Carlsson 2017) and reports of somatic symptoms are common among immigrant, but also among most general patients suffering from depression or anxiety (Kirmayer 2001).

### Limitations and Suggestions for Future Research

The results of the present study have to be considered in light of certain limitations. Firstly, it is important to note that participants reported not on refugees in general, but on their refugee *patients*, i.e. people who seek psychological help as they find themselves in troubling states. To obtain a broader picture of the mental health of refugees in Brazil, future studies are needed that focus on refugees outside of clinical settings.

Secondly, findings represent the subjective evaluations of psychologists who treat refugees in Brazil and do not represent quantifiable or generalisable data. For instance, participants might have been more likely to report on extreme cases when asked to describe their patients’ psychological suffering. However, as the first study investigating the suffering of refugee patients across Brazilian states, the results provide valuable insights into common social and mental health problems faced by this group and a base for further epidemiological and quantitative studies. Potentially lower rates of PTSD diagnosis in Brazil for example, might also result from professionals’ critical attitude towards mental health categorisation and the PTSD concept.

Finally, this study focused on psychologists’ experiences in treating heterogenous patients from various countries with diverse reasons for migration, heterogenous experiences of flight and post-migration stressors, and varying lengths of stay in Brazil. These factors and their complex interaction may play a crucial role in shaping patients’ mental health (Turrini et al. 2017). Also, even though participants were asked to report on their refugee patients, it is unclear if their descriptions only related to people with the respective official asylum status. Many of the psychologists also treated general immigrants and may not have differentiated between patients in relation to their immigration status when reporting on their suffering. This, as well as the diverse interview settings ranging from the South to the extreme North of Brazil, is likely to have impacted participants’ descriptions of their patients’ suffering (Andrade et al. 2012). However, the analysis of the data did not show any major differences in perspectives on patients’ suffering related to location of practise (Table 2), except for traumatic war experiences which were only described by psychologists in the South and Southeast. This might be explained by the fact that there are more Syrians applying for asylum in these regions (CONARE 2020). Patients in the North came almost exclusively from Venezuela, where there is a humanitarian crisis (CONARE 2020), but not a situation of war as in Syria. Further studies could explore the differences in suffering related to diverging asylum status and psychologists’ location of work in more detail.

## Conclusion

To our knowledge, this study is the first to investigate psychologists’ perspectives on the suffering of their refugee patients in diverse Brazilian states. It confirms previous findings highlighting depression and anxiety as common conditions among refugee patients (Lindert et al. 2009), but also supports concepts such as prolonged grief (Comtesse and Rosner 2019; Momartin et al. 2004) and cultural bereavement (Eisenbruch 1991) as valuable alternatives to common mental disorder classifications in refugees. Additionally, the present study provides evidence for a prevailing critical perspective on mental disorder classifications, such as the DSM-5, among psychologists in Brazil. Many of the participants thought of the suffering of refugee patients in relation to their social and political context and post-migration situation, rather than in relation to specific diagnostic categories. This reinforces the call to acknowledge the socio-political suffering of refugees (Droždek 2007; da Silva Machado, Barros and Martins-Borges 2019) and increases the importance of strategies that, in order to foster the mental health of refugees, address current stressors in post-migration settings, such as discrimination (Duden and Martins-Borges, 2021).

## Footnotes

1 Displaced people include various groups such as refugees who have been officially recognised as such, asylum seekers who are still waiting for a decision on their refugee status and people with humanitarian visas who do not fall officially under the UN definition of a “refugee”, as for instance many Haitians in Brazil. Nevertheless, the present article will refer to all of these people as refugees. Firstly, we hope this facilitates easier reading. Secondly, grouping these very heterogenous people together is based on the assumption that displaced people find themselves in a comparable predicament that is distinct from that of the general population.

2 Throughout the article, the denomination of terms of mental illnesses (e.g. “depressive disorder”) is in accordance with the respective reference from which the term was taken. Furthermore, the expression “psychological suffering” is used to allow for the inclusion of a range of experiences and mental states that are considered troubling, confusing or not perceived to be healthy by the people concerned. This expression was chosen to capture a wider scope of problems than the designation “mental disorder” would characterise.
